# The cellular function of SCAP in metabolic signaling

**DOI:** 10.1038/s12276-020-0430-0

**Published:** 2020-05-08

**Authors:** Sun Hee Lee, Jae-Ho Lee, Seung-Soon Im

**Affiliations:** Department of Physiology, Keimyung University School of Medicine, Daegu, 42601 South Korea

**Keywords:** Metabolic syndrome, Dyslipidaemias

## Abstract

Sterol regulatory element binding protein (SREBP) cleavage activating protein (SCAP) is a key regulator of SREBP maturation. SCAP induces translocation of SREBP from the endoplasmic reticulum to the Golgi apparatus, allowing it to regulate cellular triglyceride and cholesterol levels. Previous studies have shown that suppression of SREBP activation in SCAP conditional knockout mice reduced the accumulation of intracellular triglycerides, which eventually causes the development of metabolic diseases such as atherosclerosis, diabetes, hepatic steatosis, and insulin resistance. However, despite the significance of SCAP as a regulator of SREBP, its function has not been thoroughly discussed. In this review, we have summarized the function of SCAP and its regulatory proteins. Furthermore, we discuss recent studies regarding SCAP as a possible therapeutic target for hypertriglyceridemia and hyperlipidemia.

## Metabolic diseases: blocking fat and cholesterol production

The complex cellular functions of a membrane protein that influences fat and cholesterol levels have been reviewed by researchers in South Korea. The main role of this protein, SCAP, is to move certain transcription factors between organelles within cells, allowing them to regulate the synthesis of triglycerides (fat molecules) and cholesterol. Seung-Soon Im and co-workers at Keimyung University School of Medicine in Daegu reviewed studies of the numerous signalling pathways that influence SCAP activity, and of insulin-induced genes that bind to SCAP and inhibit its function. Genetic studies have shown that blocking SCAP in mice can reduce the development of diabetes, fatty liver disease and atherosclerosis. Several drugs for these metabolic conditions have been shown to indirectly inhibit SCAP activity, and therefore drugs that directly target SCAP should be a focus of future research.

## Introduction

Sterol regulatory element binding protein (SREBP) cleavage-activating protein (SCAP) plays an important role in regulating triglyceride and cholesterol levels in the body^1^. SCAP is an endoplasmic reticulum (ER) sterol-sensing protein that chaperones SREBP-1 and SREBP-2 from the ER to the Golgi apparatus^2^. In the Golgi, two proteases, site-1 protease (S1P) and site-2 protease (S2P) release the N-terminus of SREBP in a two-step proteolytic process, thereby allowing its entry into the nucleus^3^. However, cholesterol buildup in ER membranes prevents the exit of SCAP/SREBP complexes, subsequently aborting the proteolytic processing of SREBPs and leading to a decrease in the transcription of target genes^4^. Although SCAP plays an important role in the regulation of SREBP activity, intracellular fatty acid homeostasis and cholesterol synthesis, studies on SCAP are insufficient, and few review articles are available. Therefore, this review will discuss the various roles of SCAP in lipogenesis and the inflammatory response as well as newly discovered antagonists of SCAP as putative therapeutic targets for hypertriglyceridemia and hypercholesterolemia.

### Molecular features of SCAP

**Fig. 1:**
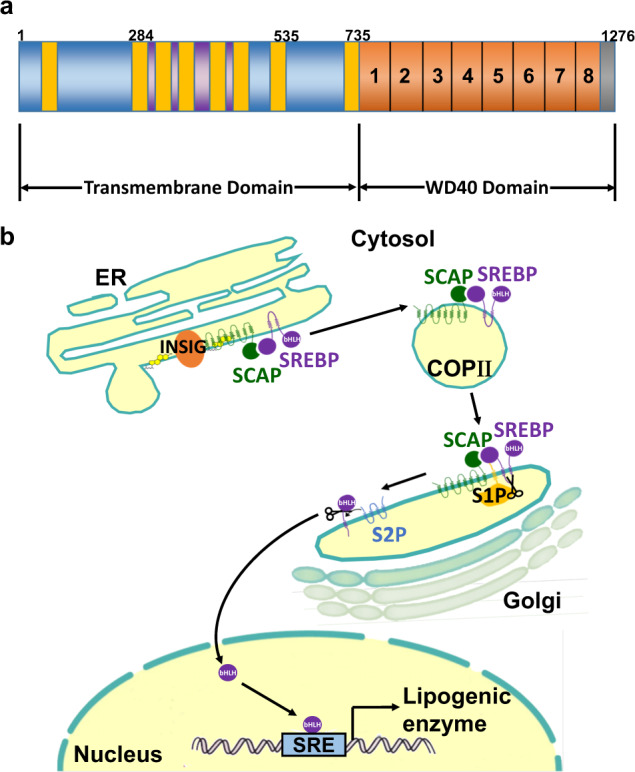
Schematic model of the domain organization and processing mechanism of SCAP. **a** SCAP proteins consist of an amino-terminal domain of eight transmembrane helices and a carboxyl-terminal WD40 domain. Transmembrane helices 2-6 of SCAP constitute a sterol-sensing domain. **b** When mammalian cells are deprived of cholesterol, SCAP escorts SREBPs in COPII vesicles from the ER to the Golgi. Two Golgi proteases (S1P and S2P) then sequentially cleave SREBPs. The NH_2_-terminal region of SREBPs moves to the nucleus and activates the transcription of target genes. To date, SREBPs are known to directly enhance the transcription of more than 30 genes needed for the uptake and synthesis of cholesterol, fatty acids, triglycerides, and phospholipids. Despite acting in diverse biosynthetic pathways, the activity of each SREBP isoform is regulated by sterols and SCAP. COPII, coat protein II; ER, endoplasmic reticulum; INSIG, insulin-induced gene; S1P, site-1 protease; S2P, site-2 protease; SCAP, SREBP cleavage-activating protein; SRE, sterol regulatory element; SREBP, Sterol regulatory element-binding protein.

SCAP (≈140 kDa) is a polytopic membrane protein composed of 1276 amino acids and can be divided into two functional regions^5^: the transmembrane N-terminal region and a soluble C-terminal domain that consists of multiple copies of a WD40 repeat motif to aid protein–protein interactions (Fig. 1a)^6^. The former region is composed of approximately 735 amino acids and functions to mediate membrane attachment^5^. It contains eight transmembrane helicases (TMs) organized into eight α-helices separated by hydrophilic loops^7,8^. These TMs are linked by four small and three large hydrophilic loops^9^. Two large rings (loops 1 and 7) are in the ER lumen, while the other large rings (loop 6) face the cytosol to combine with the coat protein II (COPII) protein to move towards the Golgi^10^. Cholesterol binding to loop 1 changes the composition of loop 6 to exclude COPII binding and prevent the exit of SCAP from the ER^9^. The latter domain, containing approximately 540 amino acids, extends into the cytosol and includes at least four WD repeat sequences that mediate its binding to SREBPs^7^. The SCAP protein forms a homotetramer with its membrane region to form a stable complex with SREBF1/SREBP1 or SREBF2/SREBP2 through its C-terminal cytoplasmic domain^11^. The translocation machinery of SCAP containing SREBP is regulated by the intracellular sterol concentration.

At high sterol concentrations, SCAP forms a ternary complex with insulin-induced gene (INSIG) via its transmembrane domains and interacts with the Sec23/24 complex in a SAR1-GTP-dependent manner through an ER export signal in its third cytoplasmic loop. Cholesterol buildup in ER membranes exceeding a threshold of 4–5% of the total lipid levels causes sterol binding to SCAP, which triggers a conformational change that, in turn, causes SCAP to bind to insulin-induced gene (INSIG) proteins (Fig. 1b)^12,13^. The addition of sterols to either intact cells or isolated membranes triggers SCAP binding to INSIGs^3^. The importance and role of INSIG were first discovered when the membrane domain of SCAP was overexpressed in cells via transfection^8^. Under these conditions, endogenous INSIGs became saturated, and sterols no longer prevented transport from the ER to Golgi^14^. When INSIGs bind SCAP, which is mediated by helices 2-6, binding of the Sec23/24-Sar1 complex is prohibited, consequently preventing SCAP from binding SREBP, resulting in suppression of movement from the ER^3^. Loop 6 of the N-terminal regions of SCAP facing the cytosol contains the hexapeptide sequence methionine-glutamic acid-leucine-alanine-aspartic acid-leucine (MELADL), which acts as the binding site for COPII proteins. The basic functional units of COPII coat proteins are Sar1, Sec23/24 and Sec13/31^15^. When sterols such as cholesterol and 25-hydroxycholesterol are used to treat cells, the lateral movement of SREBPs into COPII-coated vesicles is obstructed on ER membranes, thereby preventing SREBP maturation to suppress cholesterol synthesis^16^. To understand the molecular mechanisms by which sterols block the binding of COPII proteins to the SCAP–SREBP complex, however, it is necessary to establish an in vitro system in which this binding can be blocked by the addition of sterols to isolated membranes rather than to pre-incubated cells^16^. The feasibility of this assay is reinforced by findings that demonstrate the requirement of INSIGs, resident proteins of the ER that function as anchors, for sterol-mediated inhibition of SCAP/SREBP transport^17^. Otherwise, under sterol-depleted conditions, the SCAP/SREBP complex exits the ER by budding from the ER membranes^18^. SCAP mediates this exit using the general mechanisms defined for yeast and mammalian membrane proteins that move from the ER to the Golgi^19^.

### Roles of SCAP in lipid metabolism

**Fig. 2:**
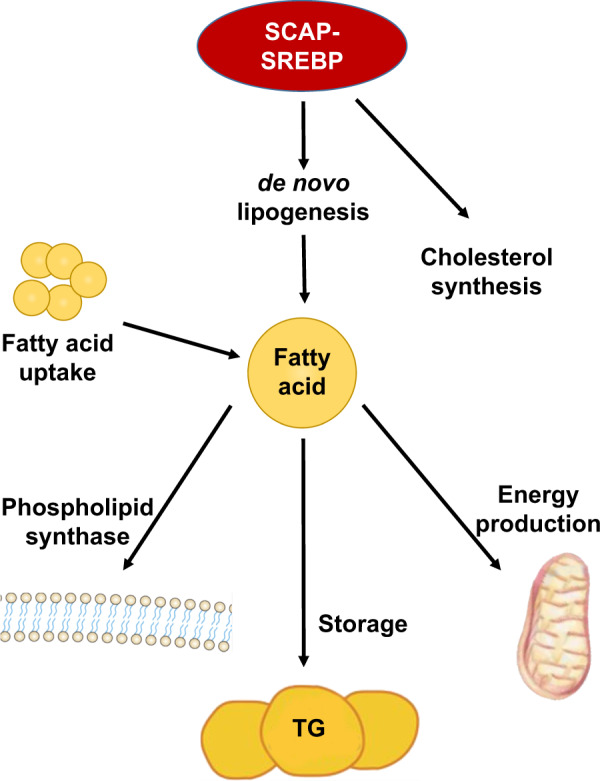
The function of SREBP-SCAP in lipid metabolism. Activation of SCAP–SREBP as the master regulator of lipid metabolism stimulates the transcription of enzymes required for de novo lipogenesis and receptors that mediate the uptake of fatty acids released by lipolysis.

SREBPs are transcription factors involved in regulating the synthesis and uptake of fatty acids and cholesterol through activating their processing mechanism by SCAP in mammalian cells (Fig. 2)^20^. In these cells, the synthesis of cholesterol and other lipids is governed by the lateral transfer of a membrane-embedded protein complex into coated vesicles, which then move from the ER to the Golgi^21^. Upon entering the nucleus, the NH_2_-terminal domains of SREBPs activate the transcription of several genes that encode proteins involved in cholesterol synthesis (e.g., 3-hydroxy-3-methyl-glutaryl-CoA synthase [HMG-CoA synthase], HMG-CoA reductase, farnesyl diphosphate synthase, squalene synthase, and others), cholesterol uptake (low-density lipoprotein receptor [LDLR]), fatty acid synthesis (acetyl-CoA carboxylase, fatty acid synthase, and stearoyl-CoA desaturase), and triglyceride synthesis (glycerol-3-phosphate acyltransferase)^2^. Sterols hinder the proteolytic cleavage of SREBP precursors, resulting in downregulated transcription of these genes^7^. Then, it allows cells to maintain a constant membrane composition despite large changes in cholesterol demand^22^.

INSIG1 and INSIG2 mediate the feedback control of lipid synthesis by sterol-dependent binding to SCAP^23,24^. The role of SCAP and INSIGs in activating SREBPs has been demonstrated in previous studies^25^. Briefly, the SCAP pathway plays a crucial role in feedback regulation of lipid metabolism and may be involved in the development of obesity. In a previous study, liver-specific loss of SCAP in high-fat diet-fed obese mice inhibited hepatic de novo lipogenesis and prevented hepatosteatosis, demonstrating the singularly important role for SREBPs relative to other nutritionally stimulated lipogenic factors^26^. An abnormal increase in de novo lipogenesis has been suggested to contribute to the pathogenesis of non‐alcoholic fatty liver disease^27^, a highly prevalent metabolic disease that is linked to the development of type 2 diabetes mellitus^28^.

### The roles of SCAP in inflammation

Cholesterol is an essential lipid in various biological processes, and the pivotal role of SCAP as a cholesterol sensor in the regulation of intracellular cholesterol homeostasis is well established^8,29^. Cholesterol deposition in dendritic cells stimulates the development of autoimmunity, possibly at the transcriptional level, through the nucleotide-binding oligomerization domain, leucine rich repeat and pyrin domain containing protein 3 (NLRP3) inflammasome^30,31^. The SCAP-SREBP2 complex promotes NLRP3 inflammasome activation, which is mainly dependent on its ER-to-Golgi translocation rather than an effect on cholesterol homeostasis. Mechanistically, NLRP3 associates with the SCAP-SREBP2 complex to form a ternary complex, which then translocates to the Golgi apparatus adjacent to a mitochondrial cluster for optimal inflammasome assembly^32^. In addition, SCAP-SREBP2 plays a role as a signaling center that integrates the inflammatory response and cholesterol metabolism in macrophages^32^. Ouyang et al. reported that the overexpression of SCAP induced cholesterol synthesis, while its knockdown reduced lipid accumulation in THP-1 human macrophages. Moreover, the overexpression of SCAP increased the levels of tumor necrosis factor α (TNFα), interleukin (IL)-1β and Monocyte Chemoattractant Protein-1 (MCP-1) production^33^. SCAP dysfunction in THP-1 human macrophages was found to affect the expression of inflammatory cytokines and lipid metabolism when the loss of SCAP significantly reduced expression levels of the TNFα, IL-1β and MCP-1 genes.

However, a controversial study demonstrated that dysfunctional SCAP stimulates an inflammatory response in THP-1 cells^34^. Suppression of the intracellular cholesterol content in THP-1 macrophages did not affect the expression of inflammatory cytokines, suggesting that the SCAP-mediated inflammatory response was independent of the regulation of cholesterol synthesis^33^. Furthermore, in a recent study regarding the molecular mechanisms of crosstalk between the inflammatory response and dyslipidemia, the loss of SCAP attenuated lipopolysaccharide-stimulated IκB phosphorylation in human macrophages and decreased the level of p65 in the nucleus, suggesting that SCAP dysfunction stimulates the inflammatory response by activating the NF-κB signaling pathway^33^. These results indicated that the function of SCAP in inflammation is independent of its role in lipid metabolism.

Inflammation disrupts the feedback regulation of LDLR to stimulate foam cell formation in macrophages^35^. SCAP recycling is a key process in the feedback regulation of LDLR and HMG-CoA reductase by controlling SCAP glycosylation in the Golgi^36^. In macrophages treated with inflammatory cytokines, mRNA and protein expression levels of mannosidase II were increased by accumulating lipid droplets and induced activation of the SREBP2/LDLR pathway^37^. SCAP glycosylation may prevent the degradation of SCAP and prolong its half-life, thereby facilitating increased SCAP recycling and activating the SREBP2/LDLR pathway.

The role of SCAP/SREBPs in the innate immune system in the context of viral, but not bacterial stimuli, was previously studied using lysozyme 2-Cre-mediated selective deletion of SCAP in macrophages (LysM-SCAP^−/−^ mice)^38^. LysM-SCAP^−/−^ mice were reported to be resistant to respiratory infection with MHV-68 and demonstrated increased expression of interferon-stimulated genes (ISGs) in the lung. As expected, cholesterol and fatty acid synthesis were reduced in SCAP-deficient macrophages. In cultured cells in which cholesterol synthesis was inhibited due to SCAP deficiency, interferon signaling was improved, and the levels of the ISGs interferon β (IFNβ) 1, myxovirus resistance (Mx)1, Mx2, and chemokine (C-C motif) ligand 2 (Ccl2) were dramatically increased^38^. Conversely, IFNβ downregulated the intracellular synthesis of cholesterol in macrophages^39^. The connection between IFNβ signaling and SCAP was further investigated using in vivo siRNAs targeting SCAP to reduce its level in the liver and leukocyte cells^40^. Compared to LysM-SCAP^−/−^ mice, an animal model treated with SCAP siRNAs was more vulnerable to viral infection and contained decreased plasma levels of IFNβ^38^. SCAP interference suppressed the expression of the ISGs, IFNα4, IFNβ1, and C-X-C motif chemokine 10 in virus-exposed macrophages^41^.

In the study on SREBP function in the adaptive immune system, SCAP was selectively deleted in T cells using Cd4-Cre (Cd4-SCAP^−/−^ mice)^42^. T cells derived from Cd4-SCAP^−/−^ mice failed to increase their levels of cholesterol and FAs after mitogen stimulation compared to wild-type mice^41^. Mitogen-stimulated SCAP-deficient T cells failed to enter the S phase of the cell cycle and thus could not proliferate. This defect was prevented by the addition of exogenous cholesterol^41,43^.

### Synthetic antagonists of SCAP

**Fig. 3:**
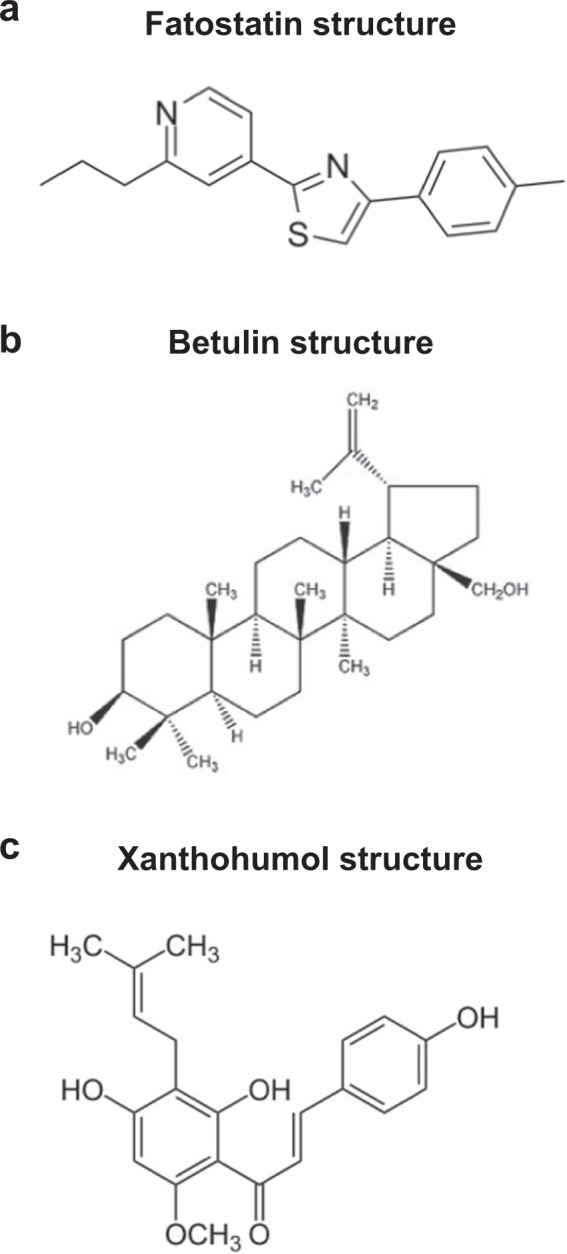
Small-molecule inhibitors of the SCAP-SREBP complex. **a** Fatostatin is a chemical inhibitor of the SREBP pathway that directly binds SCAP and blocks its ER-to-Golgi transport. **b** Betulin enhances the binding of SCAP with INSIGs, thereby promoting the retention of SREBPs in the ER. **c** Xanthohumol, a prenylated flavonoid in hops, is an antagonist of SREBP.

To date, several drugs that inhibit lipid and cholesterol synthesis have been developed^44–46^. Among them, fatostatin, betulin and xanthohumol suppress SCAP/SREBP translocation^47^. The small synthetic molecule 125B11, named fatostatin, is an organic antagonist of SCAP (Fig. 3a). By binding to SCAP^48^, fatostatin inhibits the dissociation of SCAP from INSIGs, thereby restricting the translocation of SREBPs to the ER and subsequently reducing lipogenesis and fat accumulation in obese mice^48–50^. In a previous study, fatostatin was one of the compounds that prevented insulin-induced fat production in the library of 10,000 compounds^51^. To obtain information about the specific molecular pathways affected by fatostatin, DU145 cells were treated with fatostatin, and extracted mRNA samples were analyzed by Affymetrix DNA microarray mapping of 33,000 genes^48^. Among the reduced genes, the ratios of genes associated with SCAP at high levels were analyzed. Using modified fatostatin derivatives, fatostatin was found to interact with ER proteins, and the direct interaction between SCAP and fatostatin was confirmed through binding analysis. Kamisuki et al. demonstrated that fatostatin directly interacts with an NH_2_-terminal fragment of SCAP (amino acids 1-448) including its sterol-binding domain^48^. Furthermore, previous studies have suggested that fatostatin prevents insulin-induced adipogenesis of 3T3-L1 cells^51^ and inhibits glucose-mediated activation of TGF-β in primary rat mesangial cells by inhibiting SCAP activity^34^.

Similarly, betulin binds SCAP and enhances its interaction with INSIGs to suppress SCAP/SREBP translocation (Fig. 3b)^44^. To identify small-molecule inhibitors of the SREBP pathway, various compounds were used to treat Huh7 cells^44^. Interestingly, betulin efficiently decreased the promoter activity of target genes. Furthermore, betulin stimulated the interaction between SCAP and INSIG-1, demonstrating its mechanism of action. Betulin reduces the activity of genes related to cholesterol and fatty acid biosynthesis and prevents the build-up of intracellular lipids. In diet-induced obese mice, betulin increased insulin sensitivity and reduced cholesterol and triglyceride levels^44^.

Another novel SCAP/SREBP inhibitor is xanthohumol, a prenylated flavonoid extracted from hops (Fig. 3c)^49,52^. To identify xanthohumol as a new SCAP/SREBP-inactive agent, numerous food ingredients were screened^46^. Xanthohumol was found to inhibit SREBP activity in Huh7 cells. In addition, in diet-induced obese mice, dietary xanthohumol reduced SCAP/SREBP target gene expression in the liver, thereby reducing the mature form of liver SREBP-1, which inhibited the development of obesity and hepatic steatosis. Xanthohumol inhibits triglyceride synthesis and apolipoprotein B secretion associated with the inactivation of diacylglycerol acyltransferase 1 and microsomal triglyceride transfer protein^45^. Xanthohumol interacts with Sec23/24 and blocks sorting of the SCAP/SREBP complex into COPII vesicles, thereby suppressing the ER-to-Golgi translocation of the complex^46^. Dietary xanthohumol inhibits the maturation of SREBP and transcription of its target genes^46^. Xanthohumol contributes to the amelioration of diet-induced obesity and fatty liver^53^. However, putative drugs that directly target SCAP have been still discovered.

## Concluding remarks

SCAP, a regulator of SREBP, controls the intracellular biosynthesis of cholesterol, fatty acids and triglycerides. SCAP-mediated hyperlipidemia and hypertriglyceridemia are directly related to metabolic diseases such as arteriosclerosis, obesity, and type II diabetes. These metabolic diseases secondary to high-calorie diets and hyperlipidemia are becoming a global problem. Therefore, SCAP presents a promising target for pharmacologic suppression in the treatment of metabolic diseases.
